# Hf Deposition Stabilizes the Surface Chemistry of Perovskite Manganite Oxide

**DOI:** 10.1021/acs.jpcc.0c09707

**Published:** 2021-02-08

**Authors:** Roland Bliem, Dongha Kim, Jiayue Wang, Ethan J. Crumlin, Bilge Yildiz

**Affiliations:** † Department of Nuclear Science and Engineering, Massachusetts Institute of Technology, 77 Massachusetts Avenue, Cambridge, Massachusetts 02139, United States; ‡ Department of Materials Science and Engineering, Massachusetts Institute of Technology, 77 Massachusetts Avenue, Cambridge, Massachusetts 02139, United States; § Advanced Light Source, Lawrence Berkeley National Laboratory, One Cyclotron Road, Berkeley, California 94720, United States

## Abstract

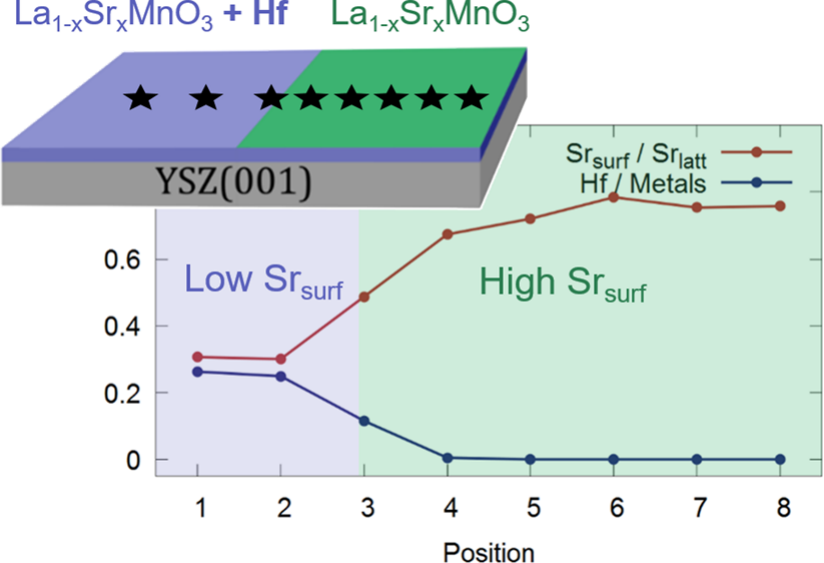

Stable composition and catalytic activity of surfaces are among the key requirements for materials employed in energy storage and conversion devices, such as solid oxide fuel cells (SOFCs). Perovskite oxides that serve as cathode in SOFCs suffer from segregation of the aliovalent substitutional cations and the formation of an inert, non-conductive phase at the surface. Here, we demonstrate that the surface of the state-of-the-art SOFC cathode material La_0.8_Sr_0.2_MnO_3_ (LSM) is stabilized against the segregation of Sr at high temperature by submonolayer coverages of Hf. The Hf is vapor-deposited onto the LSM thin film surface by e-beam evaporation. Using *in situ* near-ambient pressure X-ray photoelectron spectroscopy (NAP-XPS), we analyze the surface composition of LSM thin films. Half the LSM surface was kept as-prepared, and half was Hf-modified, for a direct comparison of untreated and Hf-treated regions on the same sample. The formation of a binary SrO_x_ surface species is quantified as descriptor for surface degradation. The onset of Sr segregation is observed at 450 °C on the bare LSM, followed by a substantial advance at 550 °C. Hf-treated regions of the same LSM surface exhibit significantly less Sr surface segregation at 450–550 °C. We interpret this stabilization imparted by Hf to arise from the suppression of the electrostatic attraction of Sr^2+^ cations to surface oxygen vacancies. Doping the surface layer with Hf, that has a higher affinity to oxygen, reduces this attraction by decreasing the surface oxygen vacancy concentration. In doing so, the use of physical vapor deposition highlights the direct role of the metal species in this system and excludes artifacts that could be introduced via chemical routes. The present work demonstrates this stabilizing effect of Hf on the surface of LSM, broadening the relevance of our prior findings on surface metal doping of other perovskite oxides.

## Introduction

Catalytic materials for energy storage and conversion have to satisfy challenging requirements, including high catalytic activity, compatibility with multicomponent materials, and long-term stability under demanding operating conditions. In applications requiring splitting, diffusion, or evolution of oxygen, perovskite oxides (ABO_3_) are considered state-of-the-art materials owing to their versatility in composition and electrical properties, structural stability, and compatibility with many other relevant compounds in energy applications.^1−3^ As a result of their desirable electrocatalytic properties, they are widely employed in devices such as solid oxide fuel cells (SOFCs),^4−10^ gas separation membranes,^11^ as well as in gas conversion, reformation,^12^ and syngas production.^13^ They are also promising for applications in advanced electronics, for example as memristive switches^14^ or in supercapacitors.^15−17^ Specifically, Mn-based perovskites present with rich chemistry, interesting physical properties, compatibility with lanthanide and alkaline earth elements, as well as high abundance and non-toxicity of their basic elements.^18−20^ Moreover, the active surface chemistry of manganites and their interaction with oxygen render them state-of-the-art cathodes for the oxygen reduction reaction (ORR) in SOFCs. A-site substituted transition metal perovskites, for example La_1–x_Sr_x_MnO_3_ (LSM), show desirable oxygen exchange kinetics^5,20−23^ and ion transport.^23,24^ The electrochemical performance of LSM as a cathode material is affected by structural and compositional variations occurring at the surface at conditions relevant to SOFC operation, for example under polarization.^25−27^ In particular, segregation and phase separation of the Sr dopant is a known problem limiting their surface stability and the rate of oxygen exchange.^28−37^ Here, we present an approach to enhance the stability of the surface chemistry of LSM at elevated temperatures. It is shown in prior work that at high temperatures in an oxidizing atmosphere, the Sr dopant cations segregate to the surface, where they accumulate and form an inert oxide layer. These Sr-rich precipitates are non-conductive (electronically and ionically)^38^ and inhibit access of gas molecules to the catalytically active perovskite surface, thus severely limiting the performance of the ORR catalyst.^31,33,36,39,40^ Targeted approaches to improve long-term stability, however, require an in-depth understanding of stable terminations, degradation processes, and interactions at the surface.^31,36,40−42^ In the case of A-site-doped transition metal perovskites related to La_1–x_Sr_x_MnO_3_, for example, a mechanistic study has identified two predominant energy terms which favor surface segregation of dopant cations.^36^ The first is an elastic energy contribution originating in the size mismatch of the dopant Sr replacing the native A-site cation La. The material can thus lower its elastic energy simply by expelling dopant cations from the La sublattice. The second is an electrostatic energy resulting from the attraction of the aliovalent dopant Sr^2+^ on La sites (Sr_La_′) to oxygen vacancies (V_O_¨) that are enriched at the surface.^36,43^ This is a more complex interaction, and depends strongly on the oxygen vacancy concentration and distribution in the material. The oxygen vacancy concentration at the surface has been predicted to exceed the bulk value by several orders of magnitude under SOFC operation conditions.^22,44^ This should result in a net attraction of negatively charged Sr^2+^ dopants (on La^3+^ sites) to the surface, which carries a net positive charge (V_O_¨). At elevated temperatures, the barriers for rearrangement and migration of ions in the lattice can be overcome, and the propensity to minimize the elastic and electrostatic energies promotes precipitation and accumulation of dopants at the surface. This results in a passivating layer and a drop in catalytic performance.^36,45−48^ The development of successful strategies to prevent this degradation has proven challenging, since the enhancement in surface stability is only helpful if the original performance as cathode can be maintained in every aspect, including catalytic activity, electronic and oxygen ion conductivity, and compatibility with the operating environment and temperatures. Our recent work addresses the segregation phenomenon by modifying the surface reducibility and thus the oxygen vacancy concentration of a model perovskite oxide via metal deposition at the surface: In the case of the intermediate-temperature cathode material La_1–x_Sr_x_CoO_3_, the deposition of Zr and Hf has been shown to clearly enhance the material’s stability against Sr segregation by decreasing the surface concentration of oxygen vacancies.^49^ This result presented simultaneously with an increase in the rate and stability of oxygen exchange reactions at the surface. In that work, the deposition of surface-modifying cations has been performed via a chemical route: by dipping the samples into an aqueous metal chloride solution. It is worthwhile to explore this surface modification also for high-temperature cathodes, such as LSM, since the surface and interface stability is equally crucial for the high-temperature mechanisms of oxygen activation and incorporation.^6,13,20^ Here, we use an alternative, ultra-clean approach, modifying the surface chemistry of LSM by e-beam evaporation in ultra-high vacuum, to deposit submonolayer coverages of Hf onto LSM. This approach gave us the ability to obtain both the bare LSM surface and the Hf-deposited LSM surface on the same thin film sample, avoiding any artifacts that could be introduced from sample preparation history. For example, variations in cation stoichiometry, atomic structure, and phase or the incorporation of hydrogen, carbon, or the deposited metal species are extremely difficult to control precisely and present as sample history-dependent artifacts in such materials. We use synchrotron-based *in situ* X-ray photoelectron spectroscopy (XPS) in an oxygen environment to assess and compare the surface chemical evolution of the bare LSM region and the Hf-modified LSM region on the same sample under the same conditions. We find that the Hf deposition enhances the stability of the LSM surface chemistry against Sr segregation at elevated temperatures in an oxygen atmosphere. Our finding establishes the doping of perovskite oxide surfaces with oxidizable cations as an approach to improve the stability of high-temperature cathode materials such as LSM. Our approach based on metal evaporation in ultra-high vacuum also ensures the direct role of the metal species by avoiding any artifacts that can arise from chemical routes.

## Methods

The measurements were performed on La_0.8_Sr_0.2_MnO_3_ thin films grown using pulsed laser deposition from a (La_0.8_Sr_0.2_)_0.98_MnO_3_ target (Sigma-Aldrich) onto single crystals of 8% Y_2_O_3_-stabilized ZrO_2_(001) (YSZ, purchased from MTI Corp). The deposition was conducted in 1.3 × 10^–3^ mbar O_2_ at a substrate temperature of 700 °C using a KrF (248 nm) laser with a fluence of 1.6J/cm^2^. After the deposition, the samples were post-annealed at 700 °C for 20 min in 0.13 mbar O_2_, followed by cooling. Before further treatment, the films were immersed in deionized water for 60 s to remove SrO_x_ surface oxide components that are formed during growth and annealing. The crystallographic orientation of the films was determined using X-ray diffraction (XRD; Cu Kα radiation, Rigaku Smartlab). The surface topography was characterized using atomic force microscopy (AFM; Veeco Metrology Dimension 3100 AFM with a Nanoscope V controller; operated in tapping mode). Metals were deposited by physical vapor deposition in ultra-high vacuum at room temperature using a Focus EFM 3T electron-beam evaporator. The deposition rate was calibrated using a quartz crystal microbalance (Sentys). A thickness of 4 Å was selected, corresponding to an equivalent coverage close to a monolayer of Hf. The measured XPS peak intensities are consistent with the calibrated coverage of 4 Å Hf. The strong absorption of photoelectrons by Hf is taken into account in the calculation of the expected intensity ratio of the photoelectron peaks (Sr 3*d* + La 4*d* for LSM, Hf 4*f* for the deposited metal) by assuming an inelastic mean free path of electrons in Hf that is shorter by a factor of 3 compared to La_0.67_Sr_0.33_MnO_3_ (at a kinetic energy of 350 eV).^50^ The term “submonolayer” is appropriate for 4 Å in the present case, since the intrinsic surface roughness of the polycrystalline oxide film leads to a higher surface area and thus a slightly lower effective Hf coverage. During deposition, one half of the LSM/YSZ samples was covered by Al foil, inhibiting metal deposition on this half of the samples. This geometry allows to directly determine the effect of metal deposition on LSM by comparing spots in neighboring regions of the same sample at equivalent temperatures and oxygen pressures. This approach of obtaining both the bare LSM and Hf-deposited LSM region on the same sample ensures a consistent temperature and oxygen pressure during the experiment and avoids potential artifacts that can arise from variations in substrate quality or sample preparation history.

The metal coverage and the evolution of the surface composition were determined using *in situ* photoelectron spectroscopy experiments at a high temperature in an oxygen environment at beamline 9.3.2 of the Advanced Light Source (ALS) (Lawrence Berkeley National Laboratory). Spectra of the Sr 3*d*, La 4*d*, Mn 3*p*, O 1*s*, and Hf 4*f* regions were acquired. The photon energies were adjusted to achieve a kinetic energy of 290 eV for all elements in order to achieve comparable mean free paths of the photoelectrons. Initially, the samples were heated to 260 °C in 1.3 × 10^–3^ mbar of O_2_ to remove residual carbon. Low-temperature reference spectra were acquired at these conditions before slowly increasing the temperature at the same constant oxygen pressure until the onset of segregation was observed. The temperature was stabilized at 450 °C, and once the Sr 3*d* peak had reached a constant state, spectra of all elements were acquired in four positions (two points on the half of pure LSM, two in the region modified by metal deposition). Moreover, a “line-scan” series of spectra was acquired in eight points connecting the regions of Hf-doped and pure LSM (step size ≈0.5 mm). Subsequently, the temperature was increased to 550 °C and spectra were acquired in one point on each half of the LSM film.

## Results

**Figure 1:**
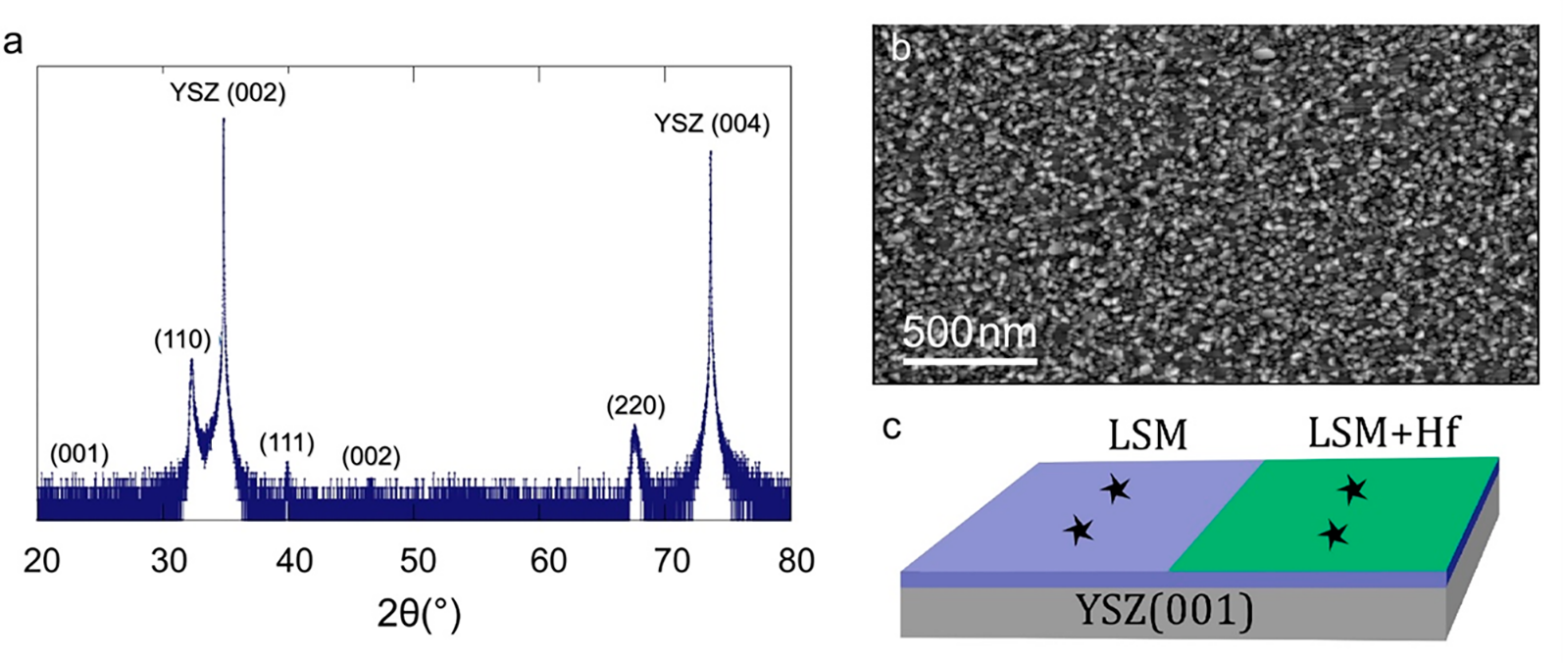
(a) X-ray diffraction pattern of as-grown La_0.8_Sr_0.2_MnO_3_ thin film on a YSZ(001) single crystal: Several low-index orientations coexist in the LSM film, dominated by the (110) plane. (b) AFM image of as-grown LSM/YSZ(001) showing a polycrystalline structure with a root-mean-square roughness of 1.2 nm (Δz = 6 nm). (c) Illustration of the sample geometry: An LSM thin film grown on YSZ(001) (10 × 10 mm^2^); half of the sample has been modified by deposition of 4 Å of Hf. The black stars indicate two measurement spots for XPS on each side of the sample.

**Figure 2:**
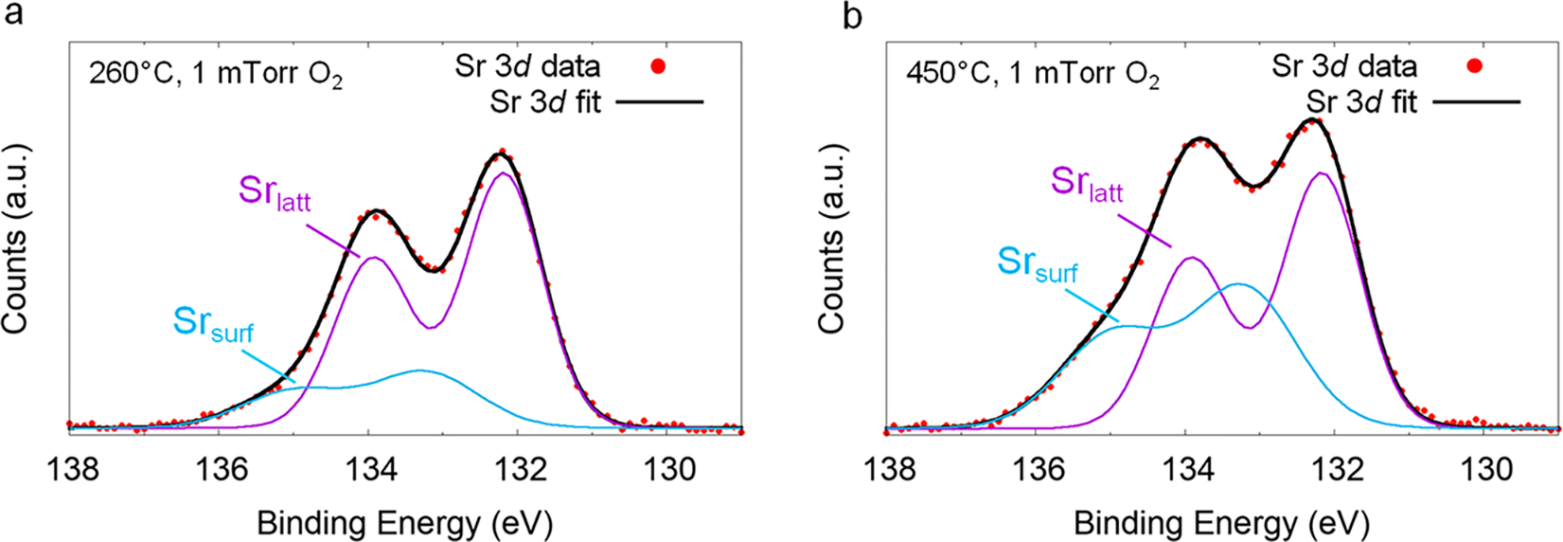
Sr 3*d* XPS spectra of La_0.8_Sr_0.2_MnO_3_/YSZ acquired *in situ* in 1.3 × 10^–3^ mbar O_2_ at 260 °C (a) and 450 °C (b). The deconvolution shows two species: Sr in the La_0.8_Sr_0.2_MnO_3_ lattice (Sr_latt_, purple) and the segregating SrO_x_ species (Sr_surf_, cyan); the sum of these two components (black line) provides an excellent fit to the experimental data (red dots). At 450 °C, a substantial increase in Sr_surf_ is observed, corresponding to surface segregation of Sr.

The surface structure and topography of the as-grown La_0.8_Sr_0.2_MnO_3_ films and the sample geometry for XPS experiments are illustrated in Figure 1. The XRD pattern of as-grown LSM/YSZ (Figure 1a) corresponds to a polycrystalline layer of LSM on single-crystalline YSZ(001), with different coexisting low-index surface orientations dominated by the (110) plane. The grain-like structure and the roughness observed in AFM images (Figure 1b, Δ*z*_max-min_ = 6 nm, r_RMS_ = 1.2 nm) are in agreement with the commonly reported columnar structure^51^ of LSM/YSZ thin films. The sample geometry for the photoelectron spectroscopy study is illustrated in Figure 1c. Hf (4 Å) was vapor-deposited onto one half of the square LSM/YSZ surface; the black stars indicate the two measurement spots on the Hf-modified and the pristine side of the sample. The deposition of Hf was not found to induce any discernible changes in the thin-film surface morphology. SrO_x_ species created already during growth at high temperature in oxygen environment, and possible carbonate species originating in the interaction with ambient atmosphere can be present in small amounts. However, formation of related overlayers or regularly occurring particulates is not discernible from AFM images. Small coverages of carbon-related species can be removed by mild oxygen annealing, which was performed as first step of the *in situ* experiments. The corresponding clean Sr 3*d* XPS reference spectrum in Figure 2a was acquired at a photon energy of 420 eV during annealing at 260 °C in 1.3 × 10^–3^ mbar O_2_. Even in the absence of carbon-related species (no discernible peak in the C 1*s* region) two components are required for a deconvolution of the Sr 3*d* region: the main component representing LSM (purple spectrum, labeled Sr_latt_) and a high-energy component indicating the presence of precipitates of segregated SrO_x_ (cyan spectrum, labeled Sr_surf_, Δ*E*_latt-surf_ = 1.04 eV).^52^ The larger line width observed of the second component is attributed to small variations in the oxygen stoichiometry of the SrO_x_ precipitates. The intensity of this second peak increases dramatically from a subtle shoulder to a defining component with annealing at 450 °C. This temperature marks the onset of significant Sr segregation, determined by monitoring the Sr 3*d* peak while slowly increasing the temperature. Changes in peak shape upon further increasing the temperature to 550 °C are subtle and not shown in Figure 2. Differences in the apparent binding energy of the Sr 3*d* region (corrected in the figure by aligning to the position of La 4*d*) and subtle variations in peak broadening between Figure 2a and b are due to charging of the sample at low temperatures, independent of the presence of Hf. This effect disappears at elevated temperatures, coinciding with an increase in conductivity of the substrate material YSZ.

**Figure 3:**
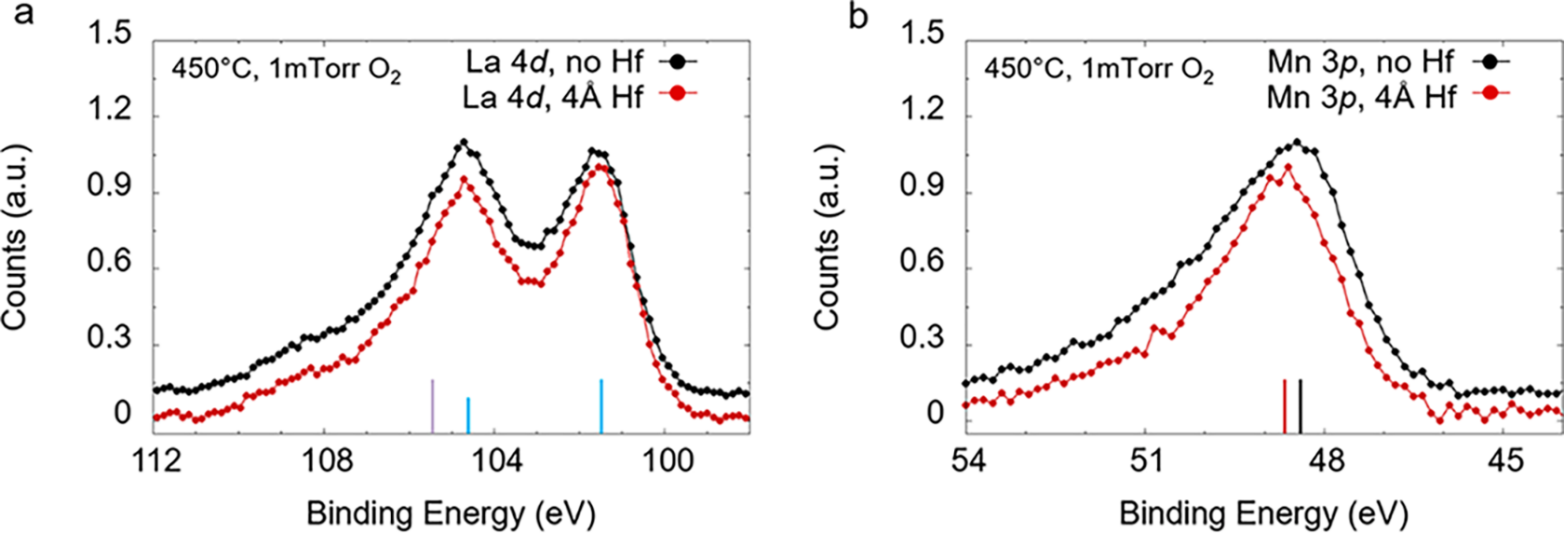
Comparison of XPS spectra of Hf-doped and unmodified La_0.8_Sr_0.2_MnO_3_/YSZ acquired *in situ* in 1.3 × 10^–3^ mbar O_2_ at 450 °C. (a) La 4*d* spectra normalized to the maximum of the 4*d*_5/2_ peak (minimum value subtracted, black curve shifted by 0.1 along the *y*-axis): In the presence of Hf, the peak maximum in the 4*d*_3/2_ region, containing a shake-up satellite, is lower than the 4*d*_5/2_ peak. The blue lines indicate the positions and ratio of the 4*d*_5/2_ and 4*d*_3/2_ peak; the purple line indicates the approximate position of the satellite. (b) Mn 3*p* spectra normalized to the peak maximum (minimum value subtracted, black curve shifted up by 0.1 along the *y*-axis). Positions of the maxima are indicated by the red and black markers. In the presence of Hf (red), intensity shifts to higher binding energies, resulting in a sharper peak at a binding energy of 48.7 eV, indicating that Mn is predominantly in a 3+ state.

The characteristic La and Mn peaks (La 4*d*, Mn 3*p*), which are close in binding energy to the Sr 3*d* core level, exhibit clear differences with the presence of Hf at the surface but remain unchanged with annealing. Representative examples for both spectral regions acquired at 450 °C in 1 mTorr O_2_ are provided in Figure 3. The difference in peak shape between the two La 4*d* spectra in Figure 3a indicates a change in the bonding environment of La with the deposition of Hf. This leads to an apparent variation in the intensity ratio between La 4*d*_5/2_ and La 4*d*_3/2_ (positions indicated by blue lines), which originates in a shift of the characteristic shake-up satellite of this core-level (Δ*E* ≈ 4 eV, purple line).^53,54^ At a higher photon energy (820 eV, Supporting Information) this effect is also discernible but weaker, emphasizing that it is localized to the surface, indicating a relation to Hf. The Mn 3*p* peak shape is also changed by the presence of Hf (Figure 3b): The spectrum of the Hf-modified side exhibits a narrower Mn 3*p* peak with a small shift of the maximum toward higher binding energy (Δ*E* ≈ 0.3 eV). The position of this distinct peak emerging with Hf doping is comparable to reference spectra for Mn^3+^,^55^ whereas the broad peak for unmodified LSM indicates the coexistence of Mn^3+^ with both lower and higher oxidation states. This difference is subtle compared to the change in La 4*d* for the highly surface sensitive spectra and not discernible at higher photon energies. The effects of Hf on both La 4*d* and Mn 3*p* remain unaffected by temperature treatment and Sr segregation. The Hf 4*f* spectrum (Supporting Information) clearly shows a single oxidized species (Hf^4+^), and remains unchanged in position and shape throughout the experiment. Its normalized intensity decreases by approximately 12% when raising the temperature to 450 °C but then remains constant at all temperatures and conditions.

**Figure 4:**
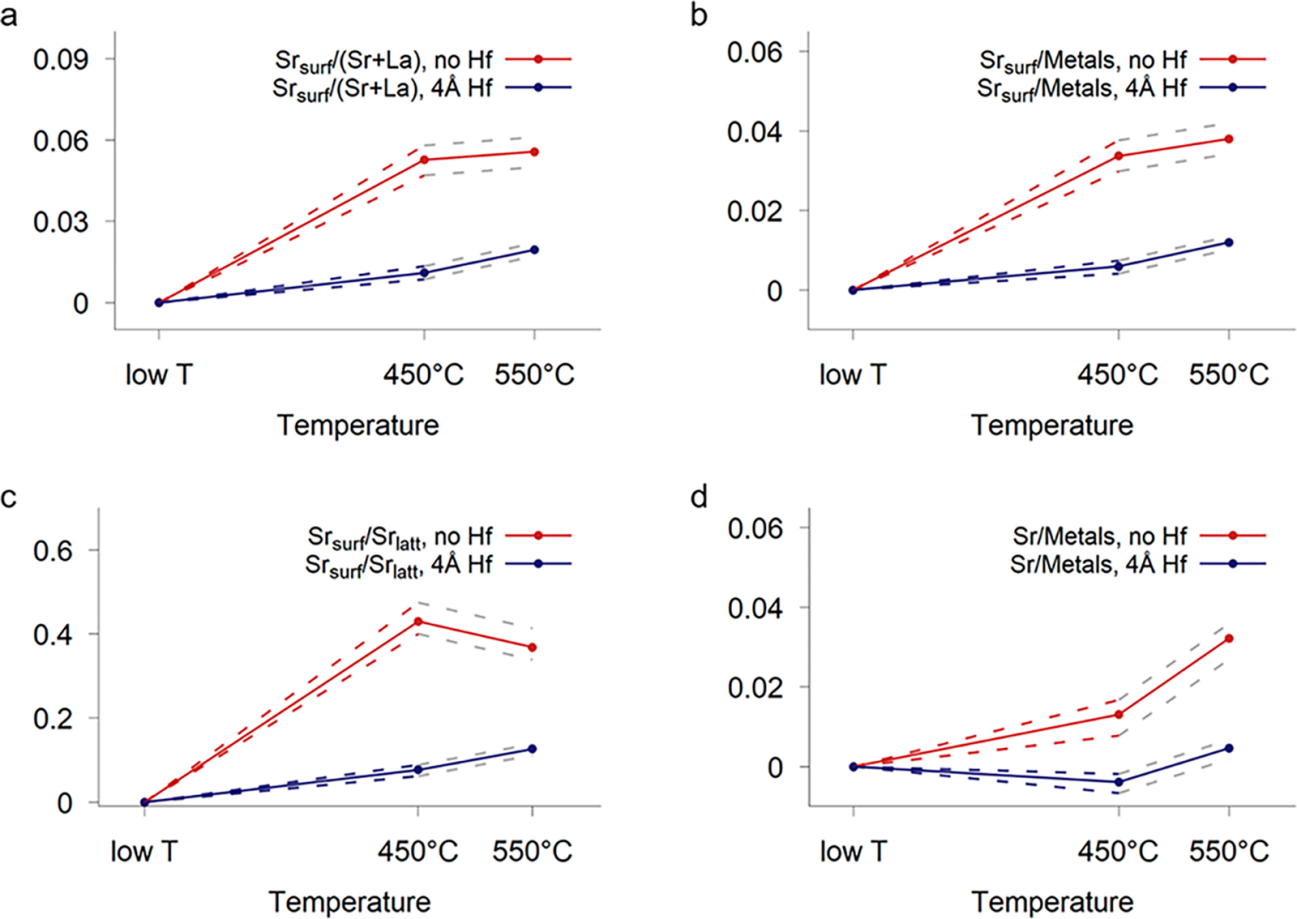
Quantification of Sr segregation on La_0.8_Sr_0.2_MnO_3_/YSZ with/without prior deposition of Hf. The intensity ratios compare the low-temperature values (subtracted) to those at 450 and 550 °C on an area with 4 Å Hf (blue) and without Hf (red) on LSM. Dashed lines indicate the maximum variation at 450 °C, continued in gray as guideline for 550 °C. The panels illustrate the temperature-dependence of (a) the ratio of segregated surface Sr (Sr_surf_) to total intensity of A-site cations, (b) the ratio of Sr_surf_ to all metals in LSM (Sr + La + Mn), (c) the ratio of segregated Sr_surf_ to Sr in the La_0.8_Sr_0.2_MnO_3_ lattice (Sr_latt_), and (d) the ratio of total Sr to the total cation intensity.

**Figure 5:**
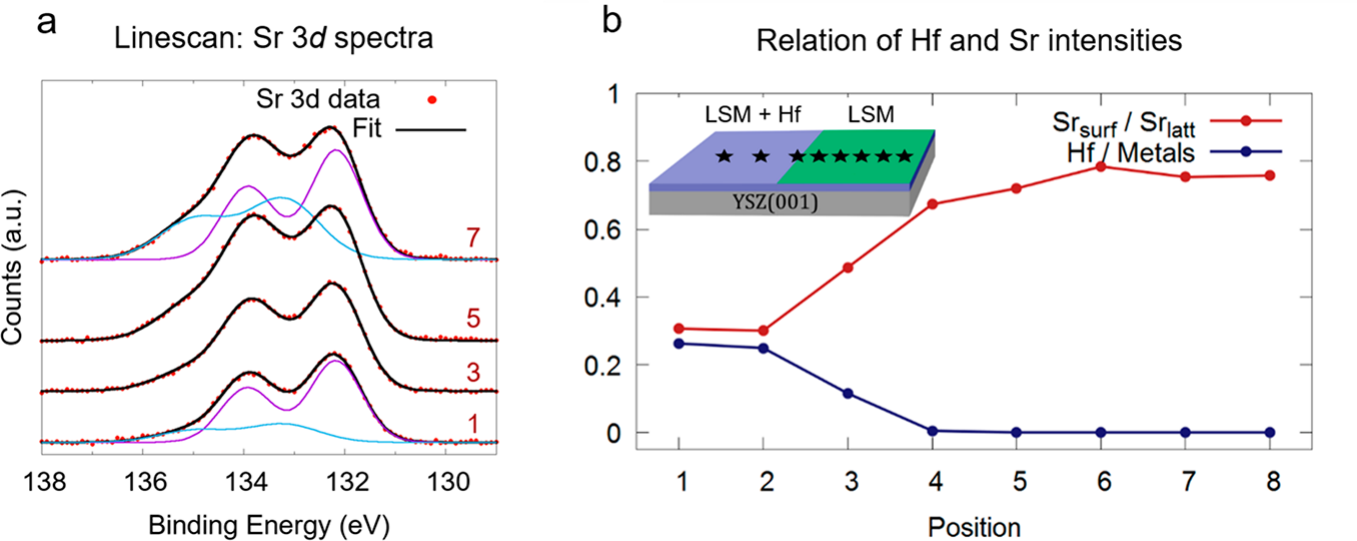
Line scan across the La_0.8_Sr_0.2_MnO_3_ thin film at 450 °C in 1.3 × 10^–3^ mbar O_2_. (a) Sr 3*d* spectra in four positions in different locations moving from the Hf-doped side (1) via a transition zone with low Hf content (3) to the undoped region (5, 7). Segregation increases with decreasing Hf content. (b) Relation of Hf content and Sr_surf_/Sr_latt_ ratio in 8 points along a linear path across the sample. Sr_surf_/Sr_latt_ shows a direct dependence on the Hf content. The schematic in the top left corner illustrates the location of the Hf modified region and the measured spots on the LSM/YSZ(001) sample.

Figure 4 displays a quantitative analysis of the contents of Sr_surf_, Sr_latt_, and total Sr that compares the Hf-doped and unmodified cases. For a clear illustration of the changes in the ratios, the low-temperature reference values have been subtracted; the graphs show the variations with high temperature. The dashed lines indicate the variations between the values acquired in different points at 450 °C. For 550 °C, the same values are used as a guideline for typical variations, indicated by the use of gray dashed lines. The peak intensities of Sr and Sr_surf_ are generally found to grow with increasing the temperature to 450 and 550 °C. This is borne out in larger contributions of segregated Sr to the total intensity of A-site cations Sr_surf_/(Sr+La) (Figure 4a) and the total intensity of all cations Sr_surf_/(Metals) (Figure 4b) as well as the ratio between surface and lattice component Sr_surf_/Sr_latt_ (Figure 4c). The ratio between the total intensity of Sr and all cations Sr/(Metals) only increases in the absence of Hf (Figure 4d). In all cases, there is a clear effect of Hf: all results related to Sr_surf_ are substantially lower in the Hf-doped case, and no clear enrichment in the overall Sr content is observed in Sr/(Metals) ratio in Figure 4d. The pronounced difference in Sr segregation between the two sides of the sample at the onset of segregation at 450 °C is also maintained at 550 °C. On the unmodified LSM surface, the higher temperature initiates a new phase in the segregation process: enrichment in both Sr-related species. While the intensity of Sr_surf_ continues to grow on both halves of the sample, the unmodified LSM surface also undergoes substantial Sr_latt_-enrichment. This is borne out in the strong increase in the Sr/Metals ratio (Figure 4d), as well as in the drop in the direct comparison of Sr_surf_ and Sr_latt_ species (Figure 4c), while Sr_surf_/(Sr+La) and Sr_surf_/(Metal) increase significantly (Figure 4a,b). After acquiring full sets of XPS spectra for the comparison at 450 °C, a detailed position-dependent analysis of the correlation of Sr segregation and Hf content was performed by subsequently acquiring spectra in eight different points moving from the Hf-doped to the unmodified side of the sample. Figure 5a shows the Sr 3*d* spectra in four of these positions to illustrate the evident change in shape owing to the growth of the high-binding-energy peak (cyan component). The position-dependent values of the Sr_surf_/Sr_latt_ ratio and of the surface Hf content show a clear correlation, which is depicted in Figure 5b: The Sr_surf_ contribution is approximately constant on the Hf-doped side of the sample but increases progressively with decreasing Hf content in the transition zone in the middle of the sample (position 3) and reaches a higher plateau on the side without Hf. It should be noted that the Sr_surf_ content on both sides of the sample remained constant within one standard deviation during more than 4 h of measurement at 450 °C.

**Figure 6:**
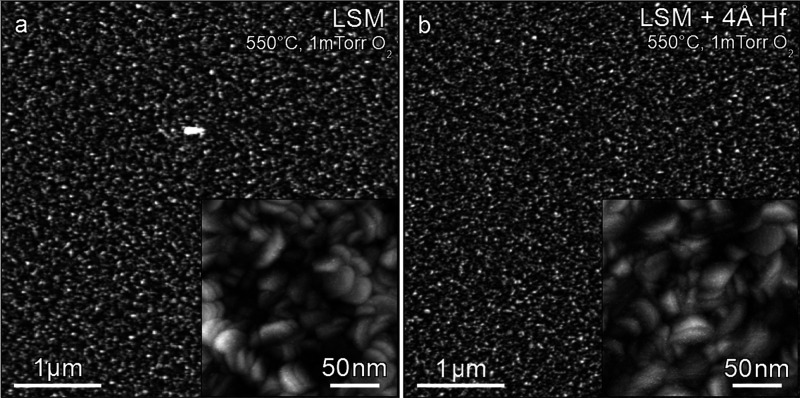
Atomic force microscopy and scanning tunneling microscopy (insets) images of La_0.8_Sr_0.2_MnO_3_ acquired after the *in situ* XPS measurements in the Hf-free (a) and Hf-doped (b) regions. Both the root-mean-square roughness (≈0.96–0.98 nm in the AFM images) and the average feature shape and size are very similar. The bright feature in the center of panel a is considered unrelated to Sr segregation; a random distribution of comparable particles is observed on the entire sample.

While the effect of Hf deposition is clear and pronounced in XPS spectra, scanning probe microscopy images (atomic force microscopy and scanning tunneling microscopy) do not show any discernible differences in the surface morphology at the resolution that could be achieved for these relatively rough surfaces (r_RMS_ ≈ 1 nm). Figure 6 shows a comparison of AFM and STM images acquired *ex situ* after the XPS experiments described above. A grainy structure with a root-mean-square roughness of ≈0.96–0.98 nm is observed in large-scale AFM images (4.5 × 4.5 μm^2^, Δ*z*_max-min_ = 5.6 nm) in points without Hf (a) and with 4 Å of Hf (b) deposited prior to the annealing experiments. The STM images in the insets (200 × 200 nm^2^, Δ*z*_max-min_ = 7.5 nm) do not reveal any discernible differences in the shape and size of the observable features. The high, bright feature in the center of Figure 6a is considered a dust particle or contaminant, since similar particles are observed in random distribution in all locations imaged on the sample. Moreover, they do not resemble Sr precipitates observed in previous reports of Sr segregation on LSM^36^ in size or density.

## Discussion

The experimental results presented above demonstrate the stabilizing role of modifying the surface of the high-temperature SOFC cathode material LSM with Hf, deposited by electron-beam evaporation. *In situ* XPS measurements at elevated temperature in oxygen environment allow us to follow the extent of Sr segregation on polycrystalline LSM films on YSZ(001) as a function of temperature and Hf coverage (Figures 4, 5) in an oxygen environment. In agreement with literature,^28,29,34,35^ a general surface enrichment in Sr is observed starting at an onset temperature close to 450 °C; in particular, the Sr_surf_ component, corresponding to an inert, segregated SrO_x_ species, starts to increase on the entire sample at this temperature. A direct comparison of regions with and without Hf demonstrates that the presence of submonolayer coverages of Hf at the surface diminishes the extent of Sr segregation. This is borne out in smaller intensity changes of the Sr_surf_ component as well as the lower overall Sr content in the surface region accessible to XPS. This trend remains unambiguous when increasing the temperature to 550 °C: While the Sr_surf_ content increases mildly on the Hf-modified part, the Hf-free half of the sample undergoes a strong increase in the total Sr content (Figure 4d), including both the Sr_surf_ and the Sr_latt_ components. As a result of the strong increase in Sr_latt_, the Sr_surf_/Sr_latt_ ratio is even found to decrease compared to the value at 450 °C (Figure 4c), despite the fact that the absolute Sr_surf_ intensity continues to grow. Clearly, this also affects the descriptors Sr_surf_/(Sr+La) and Sr_surf_/Metals (Figures 4a,b) and leads to an under-representation of the Sr_surf_ content in the comparison of the two temperatures. This temperature-dependent analysis suggests that surface doping with Hf does not fully inhibit Sr segregation but it substantially reduces its extent and shifts the regime of strong overall Sr enrichment to higher temperatures. Thus, the presence of Hf is expected to diminish the extent of surface degradation via SrO_x_ formation, while Sr enrichment in the surface region of LSM itself appears to be largely suppressed by Hf over a significant range of operating temperatures. While Hf may also have potential as a chemical getter to inhibit SrO_x_ formation, a mechanism reported for ZrO_2_,^56^ our results indicate that the presence of Hf directly affects the segregation process rather than only changing the chemical state of Sr once precipitation has occurred. Advanced theoretical models of the defect chemistry of LSM establish a relation of Sr segregation and the distribution of oxygen vacancies, holes localized on the B-site cations in a thick layer of LSM, as well as possible dopant–dopant interactions.^57^ In contrast to a model of pristine LSM, however, we expect the presence of Hf and its likely incorporation on B-sites of the perovskite lattice to alter the electronic structure and the defect chemistry of the surface region (of a thickness of several monolayers). In a possible future theoretical analysis, these variations in composition will require attention and care, in particular since essential parameters such as the oxygen vacancy formation energy and the balance of Sr dopants and holes localized on the B-site are expected to vary between the Hf-doped surface region and the stoichiometric bulk of LSM.

The characterization of local changes in the surface topography was attempted using scanning probe microscopy (Figure 6). While the XPS results paint a clear picture of the influence of Hf, the difference in segregation is not discernible in AFM and STM images acquired *ex situ* after the XPS experiments. The images, however, exclude that SrO_x_ particles of several nanometers in size form already at the early stage of segregation. For obtaining details at a smaller size scale, the grainy structure of LSM thin films on YSZ and their large corrugation provide a difficult starting point for imaging SrO_x_ precipitates at the onset of segregation. Moreover, in *ex situ* images, the modification of the surface upon contact with ambient atmosphere cannot be excluded. In particular the formation of carbonaceous species, different oxides, or hydroxides may conceal the different electronic properties of SrO_x_ species in STM images. Even though no obvious difference can be determined by scanning probe microscopy, evidence for a step-like change in segregation behavior with Hf surface doping can be derived from XPS. The position-dependent analysis of Sr segregation at 450 °C (Figure 5) illustrates the unambiguous correlation between the presence of Hf and a lower Sr_surf_ content. The intermediate level of segregation in the transition zone between the Hf-doped and unmodified sides indicates that the stabilizing effect scales with the deposited coverage in the submonolayer regime. It is possible that the surface Hf only interacts with LSM unit cells in its immediate vicinity, leading to a linear relation of its areal density and its effect on surface stability up to a saturation coverage, which is expected to remain below a monolayer of Hf. Alternatively, the limited coverage of Hf at grain boundary sites, which exhibit high oxygen vacancy concentrations,^58^ may be the bottleneck of complete stability enhancement after the required average surface density has already been reached. The possible application of our results in energy conversion devices will require establishing a balance between the enhanced stability and the modification of the electrochemical properties of LSM by Hf doping. While workable values for the polarization resistance of several layers of hafnia and zirconia deposited onto (La,Sr)(Co,Fe)O_3_ particles have been reported,^59^ the electronic structure of the cathode surface and the related ORR activity are expected to benefit from distributing a lower coverage of Hf across several layers in the surface region of a perovskite cathode.

The observed stabilization against dopant segregation via Hf deposition is not only in good agreement with our previous report on the enhancement of stability on La_0.8_Sr_0.2_CoO_3_^49^ but also demonstrates that this impact of Hf deposition is not specific to one material or deposition method. While the solution-based deposition process in ref (49) could result in undesired modifications of the perovskite surface, the present work utilizes electron-beam evaporation, minimizing the probability for chemical or structural artifacts introduced to the surface. Moreover, the high-temperature cathode material La_1–x_Sr_x_MnO_3_ exhibits intrinsically different properties and operation mechanisms compared to those of intermediate-temperature electrode materials such as La_1–x_Sr_x_CoO_3_. While they share the propensity for deactivation via dopant segregation, the general applicability of stabilization by surface doping on different types of perovskite cathodes is not obvious. This is in particular because of the different oxygen defect chemistries they accommodate.

The low coverage of Hf, vapor-deposited at room temperature, also highlights the importance of the surface region in segregation processes. Were bulk effects to dominate the dopant segregation behavior, the impact of the highly localized Hf-containing layer on the enrichment and precipitation of Sr-based species in the surface region would be negligible. The electrostatic attraction of Sr to a higher density of oxygen vacancies in the surface region^36^ is affected strongly by the surface composition, in particular by the presence of an element that modifies the surface reducibility. In the present case, Hf lowers the reducibility of the surface and thus weakens the electrostatic driving force of Sr segregation.^49^ The remaining extent of segregation in the Hf-modified region can thus be attributed mainly to the elastic driving force, which remains largely unaffected by the deposition of Hf localized to the surface, in contrast to the reported elastically dominated stabilization of SrTi_0.50_Fe_0.50_O_3_ via bulk doping with Hf and Zr.^60^ However, since the nominal Hf coverage of 4 Å is not expected to fully saturate the highly corrugated LSM surface, part of the segregation could be due to an inhomogeneous Hf distribution, leaving small patches unaffected, in particular grain boundaries and areas shadowed by large grains or particles. In the central region of the sample, where the Hf signal decreases by approximately a factor of 2, the extent of segregation increases substantially (point 3 in Figure 5b). This observation is a strong indicator that the stabilizing effect increases monotonously with the Hf coverage (still in the submonolayer regime), as one would expect for modifications of the charge states in the surface region by metal cation doping. In contrast to the electrostatic energy, the elastic properties of the LSM film remain largely unaffected, indicating that even stronger stabilizing effects may be attainable for dopant cations with an intrinsically lower elastic energy due to a smaller size mismatch to La, for example Ca.

## Conclusions

We prepared a La_0.8_Sr_0.2_MnO_3_ thin film with coexisting Hf-modified and unmodified regions using electron-beam evaporation, to study the effect of Hf surface doping on the surface stability of LSM at elevated temperatures. This configuration enabled us to compare the extent of degradation via Sr segregation on the same sample and unequivocally demonstrate the stabilizing effect of Hf on LSM. Thereby, we generalize the stability enhancement achieved via chemical deposition of Hf and Zr from liquid that was reported previously for the intermediate-temperature cathode material La_0.8_Sr_0.2_CoO_3_.^49^ The use of electron-beam evaporation excludes potential artifacts that can be introduced during the chemical deposition process and thus emphasizes the effect of the deposited metal species on the surface stabilization. Furthermore, the results demonstrate that the stabilization of perovskite oxide surfaces by Hf deposition is applicable not only to La_0.8_Sr_0.2_CoO_3_, as previously shown, but also to La_0.8_Sr_0.2_MnO_3_.

## Supporting Information Available

Figure S1: XPS spectra of the La 4*d* and Mn 3*p* regions of LSM at high photon energy (820 eV), acquired at 450 °C in 1 mTorr O_2_ with and without Hf; Figure S2: XPS spectra of the Sr 3*d* region of LSM at high photon energy (820 eV), acquired at 450 °C in 1 mTorr O_2_ with and without Hf; Figure S3: XPS spectrum of the Hf 4*f* region of modified LSM at a photon energy of 340 eV, acquired at 450 °C in 1 mTorr O_2_; Table S1: XPS fitting parameters of the Sr 3*d* spectra shown in Figure 1 (PDF)
