# Supplementary material for: Hf Deposition Stabilizes the Surface Chemistry of Perovskite Manganite Oxide
Source: J Phys Chem C Nanomater Interfaces. 2021 Feb 8;125(6):3346–54. doi: 10.1021/acs.jpcc.0c09707 (PMC8016110; doi:10.1021/acs.jpcc.0c09707)
Supplement: Supplementary file 1 — jp0c09707_si_001.pdf [file jp0c09707_si_001.pdf]

Supporting Information: Hf Deposition Stabilizes the Surface Chemistry of Perovskite Manganite Oxide

Roland Bliem[a, †,‡,*], *Dongha Kim*[b,‡], *Jiayue Wang*[a], *Ethan J. Crumlin*[c], *Bilge Yildiz*[a,b,*].

a. Department of Nuclear Science and Engineering, Massachusetts Institute of Technology, 77 Massachusetts Avenue, Cambridge, Massachusetts, 02139, USA

b. Department of Materials Science and Engineering, Massachusetts Institute of Technology, 77 Massachusetts Avenue, Cambridge, Massachusetts, 02139, USA

c. Advanced Light Source, Lawrence Berkeley National Laboratory, One Cyclotron Road, Berkeley, California, 94720, USA

† **Present affiliation**: Advanced Research Center for Nanolithography, Science Park 106, 1098XG Amsterdam, The Netherlands, and Institute of Physics, University of Amsterdam, Science Park 904, 1098XH, Amsterdam, The Netherlands

The La 4*d* and Mn 3*p* reference spectra were acquired at photon energies of 390 eV and 340 eV, respectively, but also at a higher photon energy (820 eV), corresponding to higher probing depth and thus lower surface sensitivity. The shifts in position and intensity described in the main text are also discernible at a photon energy of 820 eV but weaker (see Figure S1).

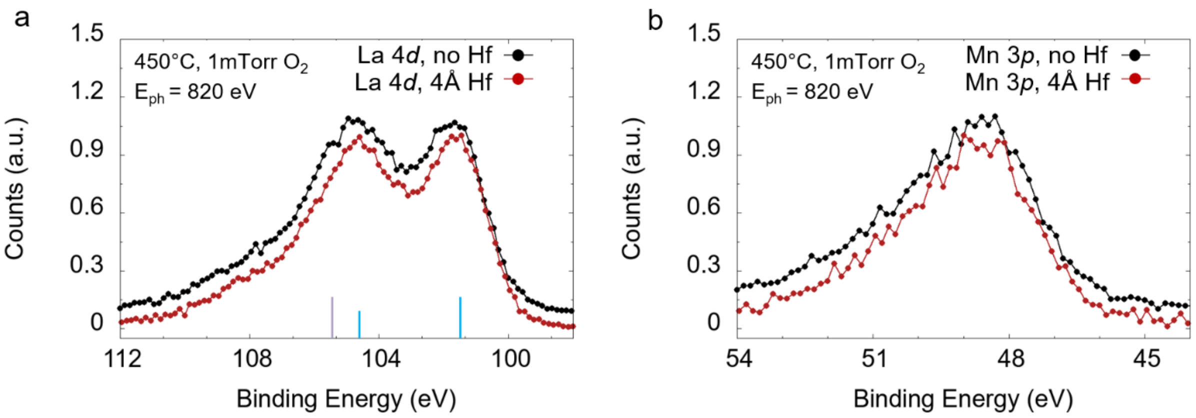

Figure S1. La 4*d* (a) and Mn 3*p* (b) XPS spectra of LSM with (red line) and without Hf (black line) acquired at 450°C and a photon energy of 820 eV (background subtracted). The spectra have been normalized to the peak maximum; the black curve has been shifted up by 0.1 along the y axis. The blue markers in (a) indicate the peak position of the La $4d_{5/2}$ and La $4d_{3/2}$; the purple line marks a typical position for a satellite peak in oxidized La compounds. The spectra do not show clear variations with Hf deposition, in contrast to the more surface-sensitive measurements presented in the main manuscript.

Similarly, XPS spectra of the Sr 3*d* levels with and without Hf were recorded at a photon energy of 820 eV, corresponding to lower surface sensitivity than the 420 eV used for the spectra presented in the main manuscript. In the direct comparison in Figure S2, a stronger flattening of the double-peak feature in the Sr 3*d* spectrum is observed for the half of the sample without Hf (black line).

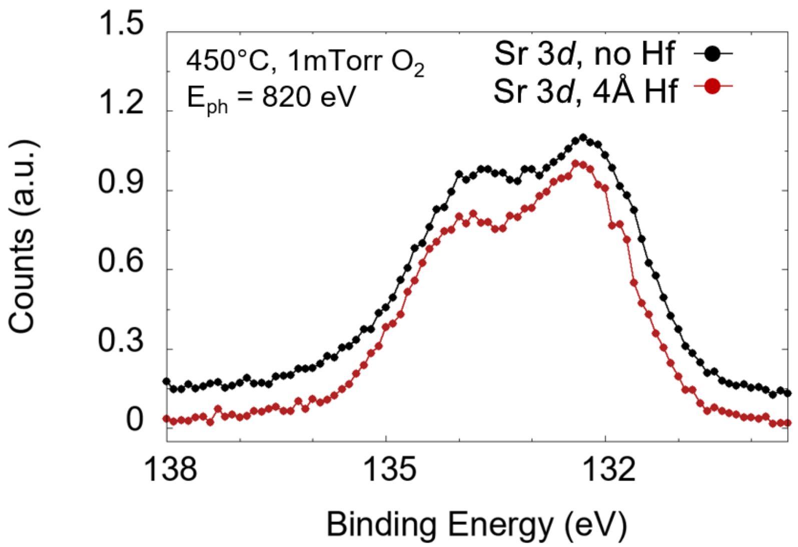

Figure S2. Sr 3*d* XPS spectra of LSM with (red line) and without Hf (black line) acquired at 450°C and a photon energy of 820 eV. The double-peak shape of the 3d level is more pronounced in the Hf-modified case, while the flattening of the peak of the black curve is indicative of an increase of the Sr_{surf} component at higher binding energies. The effect is, however, more difficult to discern compared to the more surface-sensitive spectra acquired at 420 eV.

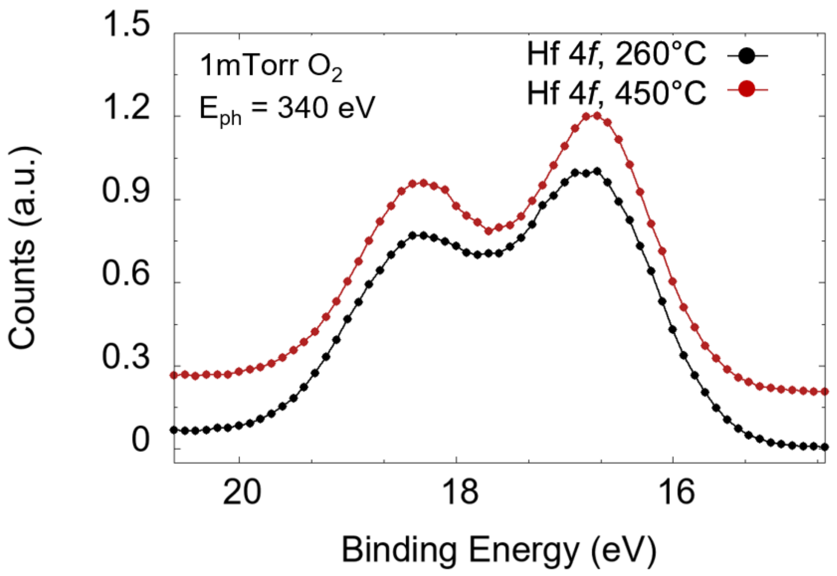

Figure S3 Hf 4*f* XPS spectra of Hf-modified LSM acquired in 1 mTorr O_2 at 260°C (black line) and 450°C (red line) at a photon energy of 340 eV. The peak is a single doublet at a peak position close to fully oxidized Hf (16.6 eV for Hf $4f_{7/2}$), and a small contribution of La 5*p* (approximately 2.5% of the peak area). The peak position has been corrected based on valence-band measurements at the same photon energy and analyzer settings.

The XPS spectra were analyzed using the peak fitting software KolXPD. All peaks were fitted using a Voigt shape of fixed Gaussian and Lorentzian line widths (Gwid, Lwid) for each experimental condition and contribution. For the Sr 3*d* spectra a detailed analysis of the contributions of Sr in the LSM lattice (lattice Sr) and segregated SrO_x (surface Sr) was performed. The detailed fitting parameters are provided in Table S1. The linewidths and shift between the lattice and surface components are provided for the spectra acquired at 450°C. All parameters were kept fixed for different positions; small variations of the Gaussian width was permitted with variation of the temperature.

Component	ΔBE	Area (raw)	Lwid	Gwid	Split
Sr_{latt}	-	387613.6	0.044 eV	1.20 eV	1.75 eV
Sr_{surf}	1.04 eV	275417.6	0.12 eV	1.59 eV	1.75 eV

Table S1. Fit parameters of the Sr 3*d* XPS spectra of LSM shown in Figure 2. The two components Sr_{latt} and Sr_{surf} represent Sr in the perovskite structure and segregated SrO_x, respectively. The different bonding environment justifies the use of different linewidths (Lwid, Gwid) for the two species. The binding energy shift (ΔBE) and split values are kept constant for all positions and temperatures.
